# New Genotypes of *Orientia tsutsugamushi* Isolated from Humans in Eastern Taiwan

**DOI:** 10.1371/journal.pone.0046997

**Published:** 2012-10-10

**Authors:** Hui-Hua Yang, I-Tsong Huang, Chin-Hui Lin, Tren-Yi Chen, Li-Kuang Chen

**Affiliations:** 1 Institute of Medical Sciences, Tzu Chi University, Hualien, Taiwan; 2 Contract Laboratory of Viral and Rickettsial Infection, Buddhist Tzu Chi General Hospital, Hualien, Taiwan; 3 Emerging Infectious Pathogen Research Laboratory, Buddhist Tzu Chi General Hospital, Hualien, Taiwan; 4 Department of Laboratory Medicine, Buddhist Tzu Chi General Hospital, Hualien, Taiwan; 5 School of Medicine of Chung Shan Medical University, Taichung City, Taiwan; 6 Emergency Medicine Buddhist Tzu Chi General Hospital, Hualien, Taiwan; University of Minnesota, United States of America

## Abstract

Scrub typhus, an acute febrile illness, is caused by the obligate intracellular bacterium *Orientia tsutsugamushi*. In our study, *O. tsutsugamushi* was rapidly detected and typed by polymerase chain reaction (PCR) and restriction fragment length polymorphism (RFLP) analysis of the 56-kDa type-specific antigen (TSA) gene. To investigate the genotypes of clinical variants of *O. tsutsugamushi*, we collected 3223 blood samples from eastern Taiwanese patients with suspected scrub typhus from 2002 to 2008. In total, 505 samples were found to be positive for scrub typhus infection by PCR, and bacteria were isolated from 282 of them. Four prototype genotype strains (Karp, Kato, Kawasaki and Gilliam) and eleven different Taiwanese genotype isolates (Taiwan-A, -B, -C, -D, -E, -G, -H, -J, -N, -O and -P) were identified by RPLF analysis. Taiwan-H, the major genotype in eastern Taiwan, exhibited prevalence and isolation rates of 47.3% (239/505) and 42.6% (120/282), respectively. We also assessed the genetic relatedness of the 56-kDa TSA gene among eight Taiwan-H isolates, thirteen other Taiwanese isolates and 104 DNA sequences deposited in the GenBank database using MEGA version 5.0 and PHYLIP version 3.66. We found that the Taiwan-H isolates formed into a new cluster, which was designated the Taiwan Gilliam-variant (TG-v) cluster to distinguish it from the Japanese Gilliam-variant (JG-v) cluster. According to Simplot analysis, TG-v is a new recombinant strain among Gilliam, Ikeda and Kato. Moreover, the Gilliam-Kawasaki cluster had the highest percentage of RFLP cases and was the most frequently isolated type in eastern Taiwan (50.1%, 253/505; 44.0%, 124/282). These findings shed light on the genetic evolution of *O. tsutsugamushi* into different strains and may be useful in vaccine development and epidemic disease control in the future.

## Introduction

**Figure 1:**
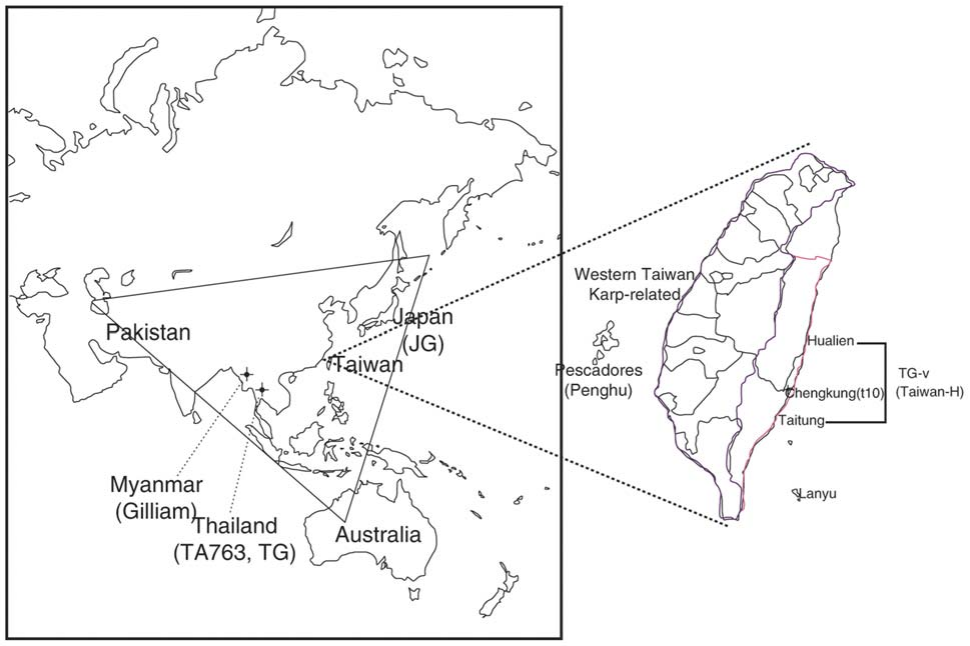
The tsutsugamushi triangle and geographical relationship of Taiwan with other countries in east Asia. Hualien and Taitung Countries are in eastern Taiwan and Chungkung Harbour is in Taitung Country. Blood samples were collected from 2002 to 2008 from patients with suspected scrub typhus in Hualien and Taitung and submitted to the laboratory for this study.

Scrub typhus (tsutsugamushi disease), an acute febrile illness, is caused by the obligate intracellular bacterium *Orientia tsutsugamushi*, which is transmitted by the bite of the trombiculid mite (or its larva, the chigger) [1]. The disease is a public health concern with approximately one billion people are at a risk of infection by the bacterium in the geographical triangle (Figure 1) extending from northern Japan in the east to Pakistan and Afghanistan in the west and northern Australia in the south [2]. Scrub typhus is a reportable infectious disease in Taiwan [3], and thousands of patients are suspected of scrub typhus infection annually. The related syndromes include meningitis, eschar, disseminated intravascular coagulation and multiorgan failure [3]. The fatality rate can reach to 50% in the absence of suitable treatment; however, scrub typhus can be treated effectively with antibiotics [4].

No effective vaccine is currently available for the prevention of scrub typhus [5], [6], [7], and vaccine development has been impeded by the lack of availability of a determinant sequence or a systematic study of *O. tsutsugamushi* serotypes and genotypes, particularly clinical variants. Six antigenic variants of *O. tsutsugamushi* have been identified previously by different groups. Shishido *et al.* reported three prototype strains for the Gilliam, Karp and Kato strains [8]. Another three prototype strains—Shimokoshi, Kawasaki and Kuroki—isolated from patients in Japan were demonstrated to belong to different antigenic types by Ohashi *et al.* [9], [10], [11]. Using the outer membrane 56-kDa type specific antigen (TSA) protein gene, several investigators in Japan, Korea, Taiwan, Thailand, Australia and China have characterized antigenic variation of *O. tsutsugamushi*, from patients, wild rodents and trombiculid mites in different endemic areas. The 56-kDa antigen is usually strain specific, but shows cross-reactivity to some monoclonal and polyclonal antibodies [9], [12], [13], [14]. Most clinical isolates are antigenically identical but some differ from their prototypes [12], [14], [15] and cannot be classified or associated antigenicly with any prototype strains. Genotyping of different clinical isolates had shown to overcome these problems [13], [14], [16], [17]. The first reported 56-kDa TSA nucleotide sequence of *O. tsutsugamushi* was that of the Karp strain [18]. Later, Tamura *et al.* used the nucleotide variation in the 56-kDa TSA gene to classify *O. tsutsugamushi* strains into the following subtypes: Gilliam [19], Japanese Gilliam (JG) [13], Karp [19], Japanese Karp type-1(JP-1) [14], Japanese Karp type-2 (JP-2) [13], [14], Kato [19], [20], Kawasaki [20], Kuroki [10], [20], Shimokoshi [20] and TW46-1 [15]. In addition, the 56-kDa TSA gene, consisting of four variant domains, has been a target for molecular detection and phylogenetic analysis [6], [12], [13], [15].

Immunofluorescent antibodies have been the traditional method to analyze the variation in the 56-kDa TSA gene of *O. tsutsugamushi* in Taiwan [12], [15], [16]. Some studies have used the polymerase chain reaction (PCR) followed by the restriction fragment length polymorphism (RFLP) analysis to further characterize nucleotide sequence variation in the gene [12], [15], [17], [21]. Six different Taiwanese genotypes, Taiwan-A, -B, -C, -D, -E and -F, were characterized by PCR-RFLP analysis of the variant domain I (VD-I) of the 56-kDa TSA gene at the Taiwan Centers for Disease Control (CDC) in 1999 [21]. Eastern Taiwan has the highest prevalence of scrub typhus in Taiwan. From 2002 to 2008, 3223 blood samples from patients with suspected scrub typhus were collected in eastern Taiwan to investigate the genotypes of clinical variants of *O. tsutsugamushi*. Nested PCR, performed by the Taiwan CDC-contracted laboratory at Tzu Chi General Hospital, was used to identify patients with scrub typhus infection. Scrub typhus infections were confirmed in 505 samples by PCR, and bacteria were isolated from 282 samples. Furthermore, the pathogen genotypes were characterized by RFLP. The 56-kDa TSA gene sequences of representative strains were examined with the aim of determining the genetic relatedness of the isolates in relation to other isolates in Taiwan or in other countries within the previously described geographical triangle [3], [6], [12], [14], [20], [21].

## Results

### PCR detection and isolation of *O. tsutsugamushi* from humans in eastern Taiwan

From 2002 to 2008, samples were collected from a total of 3223 blood eastern Taiwanese patients and subjected to PCR in this study. PCR was repeated several times with different primer pairs for the detection of *O. tsutsugamushi* infection [21], [22]. Primers tsu-34 and tsu-11 were used for the first PCR and primers tsu-10 and tsu-11 were used for the semi-nested PCR (Table 1). The molecular weights of the PCR products were 870 bp and 490 bp, respectively [22]. Of the blood samples, 505 were found to be PCR positive, and we proceeded with the PCR-RFLP assay for genotyping of the 56-kDa TSA gene. The molecular weights of the PCR products and RFLP genotypes are presented in Table 2 [21]. Bacteria were successfully isolated from 282 samples, and the RFLP genotypes of the isolated *O. tsutsugamushi* isolates are presented in Table 2. Karp, Taiwan-A and Taiwan-H were the three most commonly detected genotypes and isolates in our study, and Taiwan-H isolates were the most frequently isolates in eastern Taiwan (42.6%, 120/282).

**Table 1:** Primers used for PCR and DNA sequencing of *O. tsutsugamushi* prototype strains and clinical isolates in this study.

Primers	Primer Sequence (5′→3′)	Position*	References
Tsu-A	AGAATCTGCTCGCTTGGATCCA	147–168	[21]
Tsu-C	ACCCTATAGTCAATACCAGCACAA	578–555	
tsu-a	GAGCAGAGATAGGTGTTATGTA	263–284	
tsu-b	TAGGCATTATAGTAGGCTGA	430–411	
ST-A	TTTCG AACGT GTCTT TAAGC	(−291)–( −272)	[14]
ST-B	ACAGA TGCAC TATTA GGCAA	893–874	
ST-C	ATGCA TATAA ACCTA GCGCT	764–777	
ST-D	CTAGAAGTTATAGCGTACACCTGCACTTGC	1602–158	
Sta-A	AAATTATGTTAATTGCTAGTGCAATGTCTG	8–37	[12]
Sta-B	CTAGAAGTTATAGCGTACACCTGCACTTGC	1602–158	
tsu-34	TCAAG CTTAT TGCTA GTGCA ATGTC TGC	19–38	[22]
tsu-55	AGGGA TCCCT GCTGC TGTGC TTGCT GCG	1032–1013	
tsu-10	GATCA AGCTT CCTCA GCCTA CTATA ATGCC	408–428	
tsu-11	CTAGG GATCC CGACA GATGC ACTAT TAGGC	895–876	

* The relative position of the *O. tsutsugamushi* 56-kD TSA antigen is based on that of AF430144 (yeo-joo) and M63381 (Shimokoshi).

**Table 2:** PCR and RFLP profiles of the 56-kDa TSA gene of *O. tsutsugamushi* prototype strains and clinical isolates.

RFLP types	Endonucleases digestion (bp)			No. cases (%)	No. isolates (%)	References
	without*	*Hha*I	*Sfa*NI			
Gilliam	180	140/40	180	30 (5.9)	4 (1.4)	[21]
Karp	180	180	110/70	57 (11.2)	42 (14.8)	[21]
Kato	160	160	100/65	6 (1.2)	5 (1.8)	[21]
Kuroki	190	150/40	110/70	0	0	[21]
Shimokoshi	180	180	80/65/35	0	0	[21]
Kawasaki	180	110/60	180	2 (0.4)	0	[21]
Taiwan-A	200	200	130/70	95 (18.8)	66 (23.4)	[21]
Taiwan-B	160 (152)	160 (152)	90/70 (92/60)	4 (0.8)	2 (0.7)	[21]
Taiwan-C	160	160	160	14 (2.8)	4 (1.4)	[21]
Taiwan-D	170	70/60/40	90/80	2 (0.4)	1 (0.4)	[21]
Taiwan-E	180	180	180	3 (0.6)	2 (0.7)	[21]
Taiwan-F	?	200/150/40	150/110/70	0	0	[21]
Taiwan-G	140	140	140	16 (3.2)	10 (3.5)	In this study
Taiwan-H	170	130/40	105/65	239 (47.3)	120 (42.6)	In this study
Taiwan-J	150	150	85/65	7 (1.4)	4 (1.4)	In this study
Taiwan-N	159	159	89/71/64	15 (3.0)	12 (4.3)	In this study
Taiwan-O	190	190	83/81/27	5 (1.0)	3 (1.1)	In this study
Taiwan-P	170	170	105/65	10 (2.0)	7 (2.5)	In this study
Total (%)				505 (100)	282 (100)	

* The PCR products of the primers tsu-a and tsu-b were ranged from 140 bp to 210 bp.

### Genotypes of *O. tsutsugamushi* strains

The results of RFLP analyses of the 56-kDa TSA gene PCR products from the 505 PCR-positive and 282 isolated samples are presented in Table 2. Previously, Tamura *et al.* described six prototype genotype strains: Karp, Kato, Kuroki, Kawasaki, Gilliam and Shimokoshi [3], [10], [11], [12], [13], [17]. Six different Taiwanese genotyping isolates were also characterized by RFLP analysis by Taiwan CDC in 1999: Taiwan-A, -B, -C, -D, -E and -F [21]. In the present study, we analysed the genetic diversity of the 56-kDa TSA gene among the different cultured *O. tsutsugamushi* isolates by RFLP analyses. We detected six new RFLP genotypes among the clinical isolates cultured in eastern Taiwan: Taiwan-G, -H, -J, -N, -O and -P. However, we did not detect the three known RFLP types: Kuroki, Shimokoshi and Taiwan-F.

The molecular weights of the PCR fragments amplified from the prototype strains and clinical isolates cultured in eastern Taiwan using the primer pair tsu-a and tsu-b ranged from 140 bp to 210 bp (Table 2). The differential molecular weights of the PCR products amplified from the new Taiwanese isolates using the primers tsu-a and tsu-b were as follows: 140 bp (Taiwan-G), 170 bp (Taiwan-H), 150 bp (Taiwan-J), 159 bp (Taiwan-N), 190 bp (Taiwan-O) and 170 bp (Taiwan-P). The differential predicted restriction fragment sizes following *Hha*I digestion were 140 bp (Taiwan-G), 130 bp and 40 bp (Taiwan-H), 150 bp (Taiwan-J), 159 bp (Taiwan-N), 190 bp (Taiwan-O) and 170 bp (Taiwan-P), while those following *Sfa*NI digestion were 140 bp (Taiwan-G), 105 bp and 65 bp (Taiwan-H), 85 bp and 65 bp (Taiwan-J), 89 bp, 71 bp and 64 bp (Taiwan-N), 83 bp, 81 bp and 27 bp (Taiwan-O) and 105 bp and 65 bp (Taiwan-P). Majority of the samples shown positive by PCR were infected with Taiwan-A (18.8%, 95/505), Taiwan-H (47.3%, 239/505) and Karp (11.2%, 57/505) genotypes (Table 2).

### Phylogenetic analysis of *O. tsutsugamushi*

The molecular weights of PCR products amplified using primers ST-A and ST-D for the entire 56-kDa TSA gene of *O. tsutsugamushi* are almost 1,600 bp. The twenty-one nucleotide sequences corresponding to the 56-kDa TSA gene of the cultured *O. tsutsugamushi* isolates in this study were consecutively processed and submitted to GenBank, and their accession numbers are provided in Table 3. The genotypes of the twenty-one isolates included Taitung-1 to Taitung-7 and Hualien-1 to Hualien-14 were analyzed by the PCR-RFLP genotyping method. Taitung-1, -3 and -6 were classified as the Taiwan-A RFLP genotype; Hualien-5 and -6 were classified as the Taiwan-G RFLP genotype and Hualien-2, -7, -8, -9, -10 and -13 and Taitung-2 and -5 were classified as the Taiwan-H RFLP genotype. Hualien-1, -3, -4, -11, -12 and -14 and Taitung-4 and -7 were classified as the Taiwan-C, Taiwan-J, Taiwan-B, Taiwan-O, Karp, Taiwan-P, Taiwan-N and Taiwan-E RFLP genotypes, respectively (Table 3).

**Table 3:** Description of *O. tsutsugamushi* isolates in this study.

Isolate	Source	Location	Year of isolation	Bases	GenBank accession number	Genotype	Type by RFLP
Taitung-1	Human	Taitung	2002	1608	AF516948	Karp-related	Taiwan-A
Taitung-2	Human	Taitung	2002	1572	AY335819	TG-v	Taiwan-H
Taitung-3	Human	Taitung	2002	1608	AY357216	Karp-related	Taiwan-A
Taitung-4	Human	Taitung	2004	1605	AY787232	TA763	Taiwan-N
Taitung-5	Human	Taitung	2003	1572	AY834392	TG-v	Taiwan-H
Taitung-6	Human	Taitung	2004	1608	EF583448	Karp-related	Taiwan-A
Taitung-7	Human	Taitung	2004	1608	EU551148	JP-1	Taiwan-E
Hualien-1	Human	Hualien	2002	1557	AY243357	Kawasaki	Taiwan-C
Hualien-2	Human	Hualien	2003	1572	AY525145	TG-v	Taiwan-H
Hualien-3	Human	Hualien	2003	1590	AY636101	Kato	Taiwan-J
Hualien-4	Human	Hualien	2003	1575	AY714315	Kt-v	Taiwan-B
Hualien-5	Human	Hualien	2003	1572	AY714316	Kato	Taiwan-G
Hualien-6	Human	Hualien	2002	1572	AY714317	Kato	Taiwan-G
Hualien-7	Human	Hualien	2002	1572	AY834393	TG-v	Taiwan-H
Hualien-8	Human	Hualien	2002	1572	DQ323174	TG-v	Taiwan-H
Hualien-9	Human	Hualien	2003	1572	AY856071	TG-v	Taiwan-H
Hualien-10	Human	Hualien	2003	1572	DQ314548	TG-v	Taiwan-H
Hualien-11	Human	Hualien	2004	1605	DQ323175	TA763	Taiwan-O
Hualien-12	Human	Hualien	2004	1605	DQ323176	Karp	Karp
Hualien-13	Human	Hualien	2004	1572	DQ789360	TG-v	Taiwan-H
Hualien-14	Human	Hualien	2004	1566	DQ852664	TA763	Taiwan-P
Kato*	Human	Japan	2002	1590	AY836148	Kato	Kato
Karp*	Human	New Guinea	2002	1599	AY956315	Karp	Karp
Gilliam*	Human	Myanmar	2002	1575	DQ485289	Gilliam	Gilliam

* Kato: Taiwan CDC Kato, Karp: Taiwan CDC Karp, Gilliam: Taiwan CDC Gilliam.

**Figure 2:**
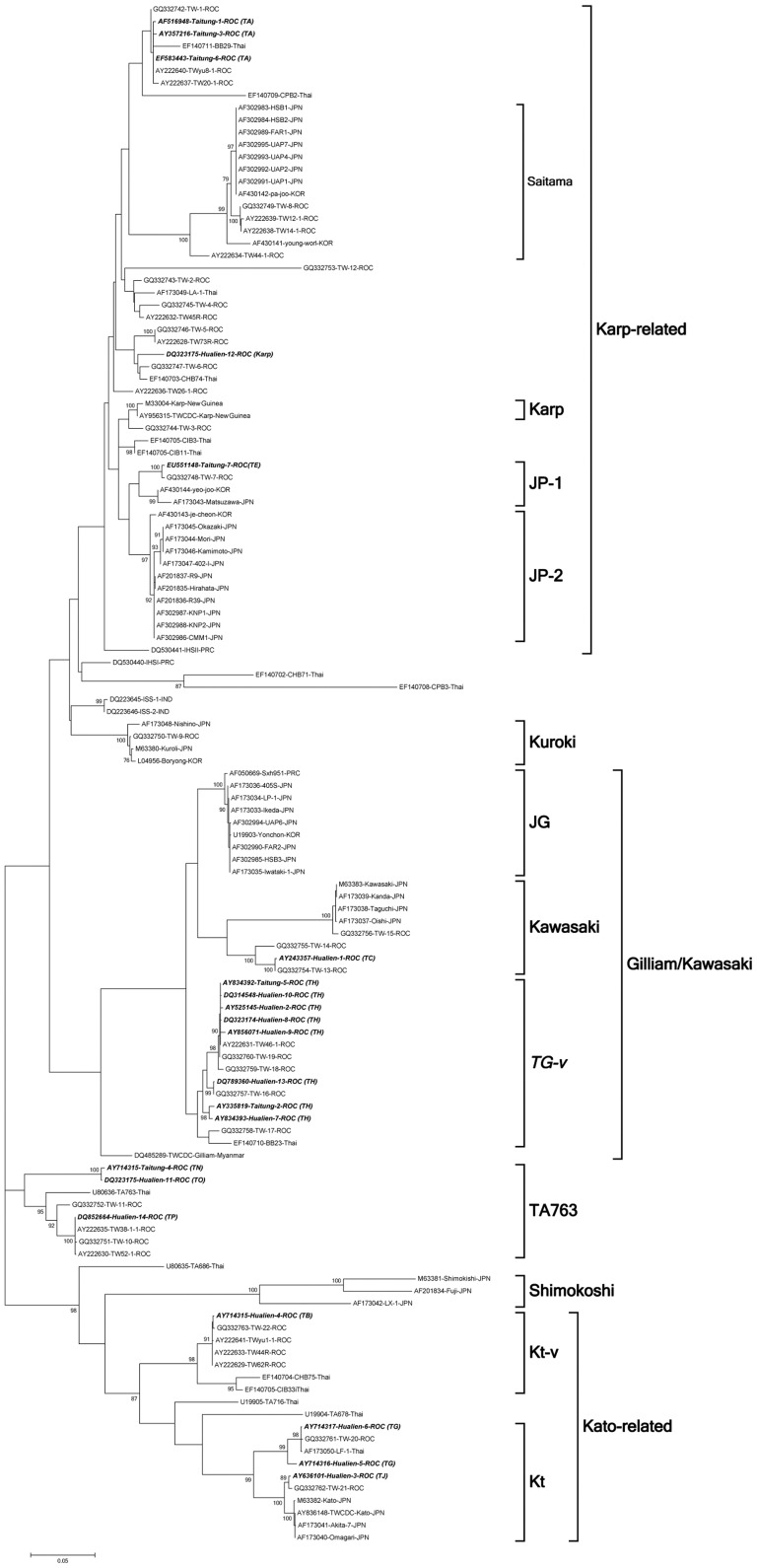
Phylogenetic tree of 125 *O. tsutsugamushi* isolates based on 56-kDa TSA gene sequences. The tree was constructed using the neighbour-joining method and MEGA software. One thousand bootstrap replicates were used to evaluate the tree reliability. Bootstrap support values greater than 75 are shown. Taiwan-H is shown in bold italics, and Taiwan Gilliam-variant cluster is enlarged in the figure S1. JP-1, Japan-Karp 1; JP-2, Japan-Karp 2; JG, Japanese Gilliam; TG-v, Taiwan Gilliam-variant; Kt, Kato; Kt-v, Kato-variant; Thai, Thailand; IND, Republic of India; KOR, Korea; JPN, Japan; PRC, People's Republic of China; ROC, Republic of China; TSA, type-specific antigen.

Using the MEGA version 5.0 and PHYLIP version 3.66 software, we further analysed the genetic relatedness of the 56-kDa TSA gene of the twenty-one isolates used in this study as well as of 104 DNA sequences previously deposited in GenBank (Table 3) [3], [4], [5], [6], [8], [14], [21], [23], [24]. The results are shown in Figure 2. The phylogenetic tree of the 56-kDa TSA gene sequences of the *O. tsutsugamushi* isolates indicated that Taitung-1, -3 and -6 cluster together in the Taiwan-A branch and that Hualien-12 belongs to the Karp branch. These four aforementioned isolates and Taitung-7 are clustered in a Karp-related gene cluster. Taitung-4, Hualien-11 and -14 were in the TA763 cluster, and Hualien-1 was in the Kawasaki cluster. Hualien-3, 4, -5 and -6 were in the Kato cluster. Hualien-2, -7, -8, -9, -10 and -13 as well as Taitung-2 and -5 are grouped in the same branch. The eight aforementioned Taiwan-H isolates were the isolates most frequently cultured from eastern Taiwan and were clustered in the Taiwan Gilliam-variant (TG-v) cluster (Figure 2 and S1). In summary, phylogenetic and RFLP analysis of the *O. tsutsugamushi* genotypes indicated that Taiwan-A, -E and Karp are Karp-related isolates; Taiwan-B, -G and -J are Kato-related isolates; Taiwan-C is a Kawasaki isolate; Taiwan-N, -O and -P are TA763 isolates and Taiwan-H isolates are grouped in the TG-v cluster. Taiwan-H (TG-v), the most common *O. tsutsugamushi* isolate in eastern Taiwan, and genotype Taiwan-P are new recombinant isolates. The similarity of the 56-kDa TSA genes of the two new isolates and other prototype strains was analysed as described in the next paragraph.

### Detection of possible recombinant isolates among various *O. tsutsugamushi* isolates and reference strains

**Figure 3:**
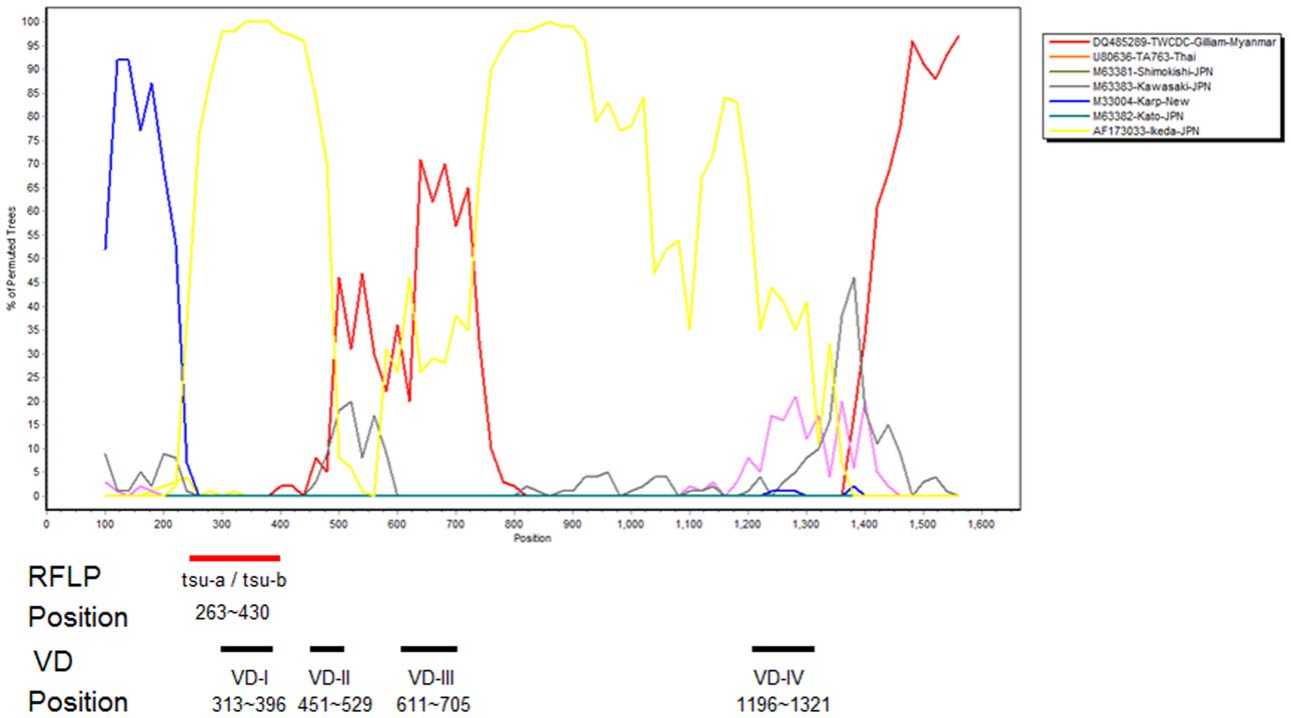
BootScan analysis of 56-kDa TSA genes of Taiwan Gilliam-variant (Taitung-5) strain and other reference *O. tsutsugamushi* strains. TSA, type-specific antigen. RFLP: tsu-a and tsu-b primer position, 263–430 (Taitung-5 nucleotides 263 to nucleotide 430); variable domain I (VD-I) position, 313–396 (Taitung-5 nucleotides 313 to nucleotide 396); VD-II position, 451–529 (Taitung-5 nucleotides 451 to nucleotide 516); VD-III position, 611–705 (Taitung-5 nucleotides 592 to nucleotide 675); VD-IV position, 1196–1321 (Taitung-5 nucleotides 1153 to nucleotide 1260).

BootScan analysis showed that the 56-kDa TSA gene sequences of eight *O. tsutsugamushi* TG-v isolates represented Gilliam, Ikeda, and Kato recombinants of identical structure, as illustrated by isolate Taitung-5 in Figure 3 [25]. A sliding window of 200 nt and a step of 20 nt were used to demonstrate the permuted tree of the 56-kDa TSA gene sequences of *O. tsutsugamushi* [26], [27], and with signals of 70% or more in the observed permuted trees indicating potential recombination events. BootScan and phylogenetic analyses showed that the 56-kDa TSA gene sequence of *O. tsutsugamushi* Taitung-5 represents a mosaic gene constructed from the 5′-terminal region (nt 1–262) and was similar to the corresponding sequence of the Kato strain (Figure S3) [28]. VD-I (variant domain I, nt 313–396), VD-IV (nt1196–1321), and the region between VD-III and VD-IV (nt 801–1195) were similar to the corresponding regions of *O. tsutsugamushi* Ikeda strain (Japanese Gilliam), and VD-II (nt 451–529), VD-III (nt 611–705) and the region of the 3′-terminal segment (nt 1322 to the end) were similar to the corresponding sequence of the Gilliam strain (Figure S3).

**Figure 4:**
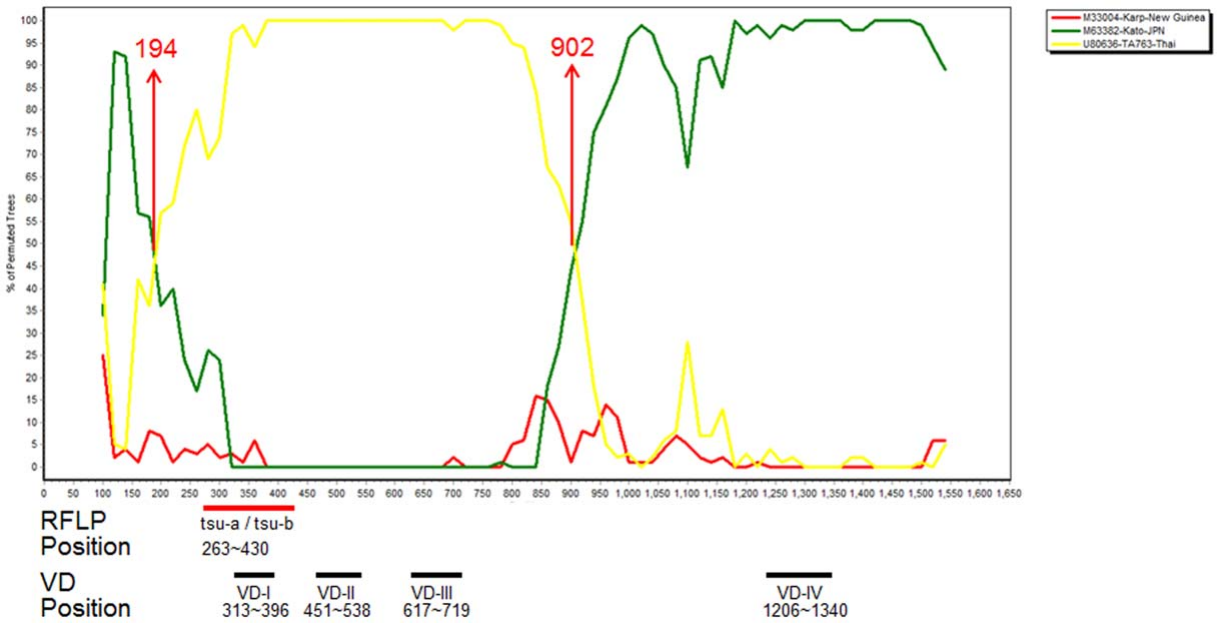
BootScan analysis of 56-kDa TSA genes of Taiwan-P (Hualien-14) strain and other reference *O*. tsutsugamushi strains. TSA, type-specific antigen. RFLP: tsu-a and tsu-b primer position, 263–430 (Hualien-14 nucleotides 263 to nucleotide 430); variable domain I (VD-I) position, 313–396 (Hualien-14 nucleotides 313 to nucleotide 396); VD-II position, 451–538 (Hualien-14 nucleotides 451to nucleotide 522); VD-III position, 598–684 (Hualien-14 nucleotides 617 to nucleotide 719); VD-IV position, 1206–1340 (Hualien-14 nucleotides 1165 to nucleotide 1257).

Based on BootScan analysis of the 56-kDa TSA gene sequences of isolates Taiwan-P (Hualien-14) and other reference prototype sequences showed that isolate Hualien-14 is a TA-763 and Kato recombinant of identical structure. We demonstrated that the isolate Hualien-14 was a recombinant originating developed from a crossover event between TA-763 and Kato strains at positions 194 and 902 (Figure 4) [27], [28]. BootScan and phylogenetic analysis of the 56-kDa TSA gene sequence of *O. tsutsugamushi* Hualien-14 with other reference strains demonstrated that the VD-IV region of the 56-kDa TSA gene sequences is similar to the corresponding part of TA763 strain, and the VD-I, VD-II and VD-III regions are similar to the corresponding sequences of the Kato strain (Figure S4) [25].

## Discussion

PCR-RFLP, multilocus sequence typing (MLST), and whole genome sequencing (WGS) are at present the most commonly used research methods in the study of bacterial evolution and classification [13], [14], [17], [21], [29], [30], [31], [32]. A series of high-throughput DNA sequencing technologies have made WGS more rapid and economical. Although WGS offers the advantage of providing complete information on the evolution of antigens and is useful in antibiotic research [31], [32], the existence of a large number of disease-causing infectious agents makes WGS more time-consuming and problematic than PCR-RFLP and MLST. MLST is a powerful method for the definitive assignment of isolates within bacterial populations to specific clones [29], [30], and is able to reveal the underlying population genetic structure. The method was used successfully in 2010 by Sonthayanon *et al.* in their study of the relationship between host infection and the rates of homologous recombination of *O. tsutsugamushi* genes [29]. Because *O. tsutsugamushi* strains exhibit antigenic diversity and can be distinguished using the sequence of the 56-kDa TSA gene, which encodes a major outer membrane protein [14], [20], RFLP-PCR analysis of the 56-kDa TSA gene is superior to MLST for immunological and vaccine studies. Furthermore, it is also more efficient than MLST analysis of seven housekeeping genes reported in the Sonthayanon *et al.* study [29].

The regions used to determine the RFLP genotypes are located in VD-I of the 56-kDa TSA gene of *O. tsutsugamushi*, and the length and restriction enzyme sites of the PCR-RFLP products vary (Table 2) [12]. Our study revealed the diversity of the 56-kDa TSA gene of the human *O. tsutsugamushi* pathogen in eastern Taiwan by RFLP analysis. The PCR method had sufficient sensitivity to detect *O. tsutsugamushi* at low levels in blood samples from infected humans. It is difficult to determine the entire 56-kDa TSA gene sequence or whole genome sequence until the bacterium is cultured. Furthermore, studies on this bacterium must be performed in expensive biosafety Level three facilities and this constraint adds to the difficulty [31], [33]. Moreover, studies are time consuming and problematic for laboratory workers. Although the entire 56-kDa TSA gene sequence is necessary for a detailed study of *O. tsutsugamushi* antigens, our study demonstrated that the PCR-RFLP genotype may be used effectively to categorize clinical scrub typhus and that the PCR-RFLP method can be useful in public health investigations of *O. tsutsugamushi*.

Our study results support the findings of previous studies. RFLP analyses of ten Taiwanese *O. tsutsugamushi* isolates in Taiwan between 1986 and 1990 have been reported by Tamura *et al.* (Figure 1) [12], [14]. RFLP revealed the ten isolates to be Karp- and Gilliam-related genotypes. The t10 isolate cultured from field rodents in Chengkung Harbour in eastern Taiwan was a Gilliam-related isolate, and it differed from the other nine Karp-related isolates cultured from other regions of Taiwan. Qiang *et al.* analyzed the 56-kDa TSA gene sequences of the ten abovementioned isolates and four additional strains isolated from Kinmen Island in 1999 [15] They found that the fourteen isolates exhibited highly variable sequences of the 56-kDa TSA gene and that TW46-1 (the t10 isolate) differed from other Karp-related isolates in the phylogenetic tree [14], [15].

Kelly *et al.* found that Karp-related isolates were the most common of all genotyped isolates by analyzing 271 sequences of the 56-kDa TSA gene at a global level [6]. Similar results were found in an analysis of 56-kDa TSA gene sequences of twenty-two *O. tsutsugamushi* isolates (TW-1 to TW-22) cultured from blood samples of scrub typhus patients in western Taiwan in 2006–2007 [3]. In our study, the distribution of RFLP genotypes and genotyped isolates in eastern Taiwan differed from that in western Taiwan and worldwide, in that Taiwan-H was the most common genotyping isolates in eastern Taiwan (Table 2). Our study revealed the geographic distribution of *O. tsutsugamushi* and correlation of genetic diversity of the 56-kDa TSA gene among eastern Taiwanese, southeast Asian, or northeast Asian isolates (Figure 1).

Taiwan-H was clustered in the same branch of the phylogenetic tree as TW46-1 from Taitung Country in eastern Taiwan, and it exhibited high sequence similarity with other isolates from Thailand (UT125, UT144, UT196 and UT396) reported by Blacksell *et al.*(data not shown) [34]. These results indicated that the cultured Taiwan-H isolates were closely related to Gilliam strain in Myanmar or to other strains in southeast Asia. The Taiwan-H cluster was designated TG-v (Taiwan Gilliam-variant) cluster in this study to distinguish it from the Japanese Gilliam-variant (JG-v) cluster named by Kelly *et al* [6]. According to the geographic distribution of *O. tsutsugamushi*, TG-v, Kawasaki and TA763 cluster isolates were found mostly in eastern Taiwan (Hualien and Taitung), and Karp-related, Kuroki and Kato-related cluster isolates were found mostly in other parts of Taiwan (Figure 1). TG-v, Gilliam, Kawasaki and TA763 cluster isolates were more closely related to isolates from Thailand and Myanmar (southeast Asia), and Kuroki, Karp-related and Kato-related cluster isolates in Taiwan were more closely related to strains from Japan, Korea and China (northeast Asia).

Eight isolates were sampled randomly from among 239 PCR-positive samples of the Taiwan-H RFLP genotype, and the full-length 56-kDa TSA gene sequences were consecutively sequenced and analyzed. The percentage of similarity to the 56-kDa TSA gene sequences among the eight Taiwan-H strains exceeded 98%, and Taiwan-H was classified as a new cluster by phylogenetic analysis (Figure 2 and Figure S1). Based on phylogenetic tree analysis of the 56-kDa TSA gene, we deduced the DNA sequences of predicted PCR products using primers tsu-a and tsu-b from the TG-v cluster of *O. tsutsugamushi* isolates TW-16, TW-17, TW-18, TW-19, TW 46-1 and BB23 isolates, which had the same *HhaI* and *SfaNI* restriction enzyme sites. The other six *O. tsutsugamushi* isolates were grouped with eight Taiwan-H genotype isolates in the same TG-v cluster by phylogenetic tree analysis. The predicted PCR product using primers tsu-a and tsu-b and RFLP analysis corresponded to the Taiwan-H genotype (Figures S1 and S2).

TG-v displayed the highest percentage of RFLP and was the most frequently isolated type in eastern Taiwan (47.3%, 239/505; 42.6%, 120/282). BootScan analysis of the 56-kDa TSA gene demonstrated that TG-v was a new mosaic isolate derived from the Gilliam, Ikeda and Kato strains (Figure 3 and Figure S3). The geographical distribution of *O. tsutsugamushi* shows that the TG-v 56-kDa TSA gene originated in southeast and northeast Asia (Figure 1). However, the genetic diversity of the TG-v 56-kDa TSA gene corresponded to Taiwan's location in the center of the geographical triangle (Figure 1). For the same reasons, the geographical distribution of *O. tsutsugamushi* showed that the origin of 56-kDa TSA gene of the Taiwan-P was TA-763 from southeast Asia and Kato from northeast Asia (Figure 4 and Figure S4).

RFLP is a rapid and powerful tool for categorizing *O. tsutsugamushi* from blood or other specimens of scrub typhus patients [14], [21]. Taiwan is located in the centre of the tsutsugamushi triangle [2], and the great diversity among *O. tsutsugamushi* pathogenic isolates found in eastern Taiwan has previously been described. Our protocol offers the advantages of using small sample amounts and avoiding the need to culture the organism before genotyping. Typing of the 56-kDa TSA gene by PCR-RFLP is valuable for classifying *O. tsutsugamushi* isolates as well as for determining the genetic relationship and epidemic evolution of *O. tsutsugamushi* isolates.

Investigations using the 56-kDa TSA gene of *O. tsutsugamushi* variants could provide much valuable information for vaccine development [5], [6], [7]. A clinical serological study showed that 20% of scrub typhus patients infected by Taiwan-H are anti-Karp antibody positive (data not shown) and that Karp or Karp-related strains may not provide immunological protection as vaccine strains if they are not used together with Taiwan-H isolates. Our findings provide a powerful method to classify the genotypes of *O. tsutsugamushi* and new insights into genetic and epidemic evolution among Taiwanese *O. tsutsugamushi* isolates, and this method may be useful in vaccine development and epidemic disease control in the Pan-Pacific region in the future.

## Materials and Methods

### Ethics statement

Ethical approval for this study was granted by the Buddhist Tzu Chi General Hospital Research Ethics Committee (IRB101-13). Scrub typhus is a notifiable infectious disease in Taiwan. The ethical standards and laboratory diagnosis of scrub typhus were based on regulations of the Taiwan CDC.

### Sample collection

Heparinised venous blood samples collected 2002 to 2008 from from eastern Taiwanese patients with suspected scrub typhus were submitted to the Taiwan CDC-contracted laboratory at Tzu Chi General Hospital for laboratory diagnosis. Peripheral blood mononuclear cells (PBMC) were separated from whole blood samples by gradient centrifugation using the Ficoll-Hypaque (GE Healthcare Bio-Sciences Corp NJ USA) technique. The samples were examined for scrub typhus positivity by nested PCR. Bacterial DNA samples extracted from PBMC of patients confirmed by Taiwan CDC-contracted laboratory to have scrub typhus infection were used in this study. The detailed procedures are described below.

### DNA extraction and PCR amplification

Bacterial DNA was extracted by using the Macherey-Nagel NucleoSpin® Blood DNA Mini Kit (Macherey-Nagel GmbH & Co. KG, Düren, Germany) according to the manufacturer's instructions and stored at −80°C. The primers used for molecular detection, PCR-RFLP genotyping, and nucleotide sequencing are shown in Table 1. Primers were designed using the framework of the 56-kDa TSA gene of the *O. tsutsugamushi* Karp strain and recognized sequences located in the conserved region. They were able to detect the prototype strains Karp, Gilliam, Kato, Shimokoshi, Kawasaki, and Kuroki as well as other isolates from the geographical triangle [17]. We used BLASTN to verify that all primers were capable of detecting *O. tsutsugamushi* genotype isolates, and the primers used are described in the next paragraph.

Primers tsu-34 and tsu-11 were used for detection during the first PCR of the clinical samples to identify scrub typhus infection [22], and the first PCR products were purified using Macherey-Nagel PCR Clean-up Gel Extraction NucleoSpin® Extract II (Macherey-Nagel). The primers tsu-10 and tsu-11 were used to improve the detection sensitivity for detection during semi-nested PCR of the first PCR products [22]. PCR amplification was performed in 50-µL volumes using the FastStart High Fidelity PCR System (Roche GmbH, Mannheim, Germany) according to the manufacturer's protocol. The reaction mixture contained 0.2 µM of each of the primers, 0.5 mM of deoxyribonucleotide triphosphate mixture and 2.5 U of FastStart *Taq* DNA polymerase (Roche). Specific fragments were amplified using one cycle of 5 min at 94°C; 40 cycles of 30 s at 94°C, 45 s at 55°C and 1 min at 72°C and one cycle of 10 min at 72°C. After electrophoresis, gels were stained with ethidium bromide and amplicons were visualized using an ultraviolet transilluminator (UVP BioImaging Systems, Cambridge, UK). The molecular weights of the PCR products were calculated using the LabWorks™ Analysis Software UVP versions 3.0.02.00 software (Solutions for Science of Life) and the results are showed in the Table 2.

### RFLP analysis

When blood samples were found to be positive for scrub typhus by PCR, the genotypes were analysed successively by PCR-RFLP. The primers Tsu-34 and Tsu-11 were used for detecting PCR-RFLP products in the clinical samples to identify the scrub typhus RFLP genotypes, and the primers tsu-a and tsu-b were used for nested PCR [21], [22]. The PCR products were purified using Montage® PCR Centrifugal Filter Devices (Millipore Corporation, Billerica, MA, USA). Specific fragments were amplified using one cycle of 5 min at 94°C; 40 cycles of 30 s at 94°C, 45 s at 55°C and 1 min at 72°C and one cycle of 10 min at 72°C. The respective nested PCR products of different *O. tsutsugamushi* genotypes are shown in Table 2. Kawamori *et al.* digested the products of nested PCR using six restriction endonucleases (*Hha*I, *SfaN*I, *Mnl*I, *AlwN*I, *Bgl*II, and *Mbo*I) and distinguished six prototype genotype strains (Karp, Kato, Kuroki, Kawasaki, Gilliam and Shimokoshi) using *Hha*I and *Sfa*NI [17]. *Chen et al.* used these two restriction endonucleases to identify six new Taiwan-specific genotypes isolates (Taiwan-A, -B, -C, -D, -E, and -F) [21]. For RFLP analysis, the purified PCR products were digested individually with *Hha*I or *Sfa*NI (New England Biolabs, USA) [21] at 37°C overnight. The *Hha*I reaction mixture contained 10 mM potassium acetate, 20 mM Tris-acetate, 10 mM magnesium acetate, 1 mM dithiothreitol, and 100 µg/mL BSA, and the *Sfa*NI reaction mixture contained 100 mM sodium chloride, 50 mM Tris-HCl, 10 mM magnesium chloride, and 1 mM dithiothreitol. The digested products were electrophoresed on 6% NuSieve agarose gels (Molecular Station, USA) as markers. The gels were stained with ethidium bromide, and DNA fragments were visualized using an ultraviolet transilluminator (UVP Biomaging Systems). The molecular weights of the PCR-RFLP product fragments were calculated using the LabWorksTM Analysis Software UVP versions 3.0.02.00 software (Solutions for Science of Life) and the results are shown in the Table 2.

### Cell culture and isolation of *O. tsutsugamushi*

L929 mouse fibroblast cells (ATCC CCL 1 NCTC Clone 929) purchased from Bioresource Collection and Research Center (Food Industry Research and Development Institute, Taiwan) were cultured in MEM containing 10% fetal bovine serum (Biological Industries, Israel) in T-75 flasks. Peripheral blood mononuclear cells were collected from acute-phase blood containing heparin (10 U/mL) and used to isolate *O. tsutsugamushi* in the Taiwan CDC-contracted laboratory; the samples were derived from scrub typhus patients. Bacterial isolates from cell culture were propagated in a T-25 flask containing a monolayer of confluent L929 cells grown in maintenance medium (MEM supplemented with 1% fetal bovine serum) at 37°C in a 5% CO_2_ incubator for 10–14 days and then detected by PCR and the indirect immunofluorescence assay; the results were confirmed by Taiwan CDC. After 14–20 days of incubation, bacteria-infected L929 cells were scraped off and stored at −80°C until use. The cultured *O. tsutsugamushi* isolates were collected from the Taiwan CDC-contracted laboratory and used in this study.

### DNA sequencing

Primers ST-A and ST-D were used for PCR amplification of the entire 56-kDa TSA gene of *O. tsutsugamushi* [14]. The PCR products were purified using the High Pure PCR Product Purification Kit (Roche) and cloned into the TA vector plasmid DNA using the pGEM®-T Easy Vector Systems kit (Promega, WI, USA). Plasmid DNA sequencing of the entire length of the cloned 56-kDa TSA gene was performed using the ABI Prism 3700 DNA sequencer (Applied Biosystems, CA, USA). Two or three different plasmid DNAs of the same *O. tsutsugamushi* isolate were sequenced to determine the accuracy of sequencing of the 56-kDa TSA gene [12], [14].

### GenBank submission

Sequences of the entire 56-kDa TSA gene of *O. tsutsugamushi* isolates from eastern Taiwan were determined and deposited in DDBJ/EMBL/GenBank databases (http://www.ncbi.nlm.nih.gov/genbank). The GenBank nucleotide sequence accession numbers for the nucleotide sequence data generated in our study are presented in Table 3. The accession numbers of the gene sequences used in our study are presented in Figure 2.

### Phylogenetic analysis and BootScan analysis

The nucleotide sequences were aligned using the ClustalW software. Phylogenetic and molecular analyses were performed using the MEGA version 5.0 [24] or PHYLIP version 3.66 software [23]. Phylogenetic trees were constructed from DNA sequences using the neighbour-joining and maximum likelihood methods, and genetic distances were calculated using the Kimura two-parameter distance algorithm. Phylogenetic relationships among the study and reference sequences were evaluated to estimate the node reliability of the phylogenetic trees constructed using the two methods, with 1000 bootstrap replicates. Bootstrap support values above 75 were considered significant.

A similarity plot was generated using SimPlot version 3.5.1 software which compares one 56-kDa TSA gene sequence from eastern Taiwan isolates used as query sequence against the 56-kDa TSA gene sequences from other strains used as reference sequences [25]. Sequences indicating possible recombination sites and crossover evens were identified sequentially. Taitung-5 (an isolate with genotype Taiwan-H) was used to investigate the distribution of recombinant forms in seven other *O. tsutsugamushi* strains (Gilliam, Karp, Kato, Kawasaki, Shimokoshi, Ikeda, and TA763). When aligning each query sequence of the above isolates to the reference isolate, it was possible to assign their particular genotypes unequivocally. The next step was the identification of closely spaced shifts among different genotype strains using the sliding window technique [26], [27], [28]. Segments of sufficient length for phylogenetic analysis and those that resulted in phylogenetic trees showing distinct clades with bootstrap values of 70% or greater were considered sufficient for subtype assignment [26], [27], [28].

The same analysis steps were performed for the Taiwan-P isolate. Hualien-14 (Taiwan-P) was examined against seven other recombinants *O. tsutsugamushi* strains of identical structure (Gilliam, Karp, Kato, Kawasaki, Shimokoshi, Ikeda, and TA763). Three neighboring *O. tsutsugamushi* strains (Karp, Kato, and TA763) were used as reference strains in order to remove excess sequence noise in the BootScan analysis. Segments of sufficient length were evaluated using 1000 Bootstrap replicates [26], [27], [28].

## Supporting Information

Figure S1 **Phylogenetic tree of Taiwan Gilliam-variant cluster based on 56-kDa TSA gene sequences was constructed using a phylogenetic tree of 125** ***O. tsutsugamushi*** **isolates.** TW-16, TW-17, TW-18, and TW-19 are from western Taiwan, TW46-1 is from Taitung Country, and BB23 is from Thailand. The percentage of similarity of the 56-kDa TSA gene sequences among the eight Taiwan-H strains exceeded 98%.
(TIF)

Figure S2 **Predicted restriction enzyme sites of the PCR product with primer tsu-a and tsu-b from the TG-v cluster of** ***O. tsutsugamushi*** **isolates.** The tsu-a and tsu-b: primer pairs, *HhaI* and *SfaNI* : restriction enzymes used in this study.
(TIF)

Figure S3 **Phylogenetic neighbour-joining trees of different fragments of 56-kDa TSA genes for a selected** ***O. tsutsugamushi*** **isolate (Taitung-5) and other reference strains.**
(TIF)

Figure S4 **Phylogenetic neighbour-joining trees of different fragments of 56-kDa TSA genes for a selected O. tsutsugamushi isolate (Hualien-14) and other reference strains.**
(TIF)
