# Supplementary figures and images for: New Genotypes of Orientia tsutsugamushi Isolated from Humans in Eastern Taiwan
Source: PLoS One. 2012 Oct 10;7(10):e46997. doi: 10.1371/journal.pone.0046997 (PMC3468442; doi:10.1371/journal.pone.0046997)

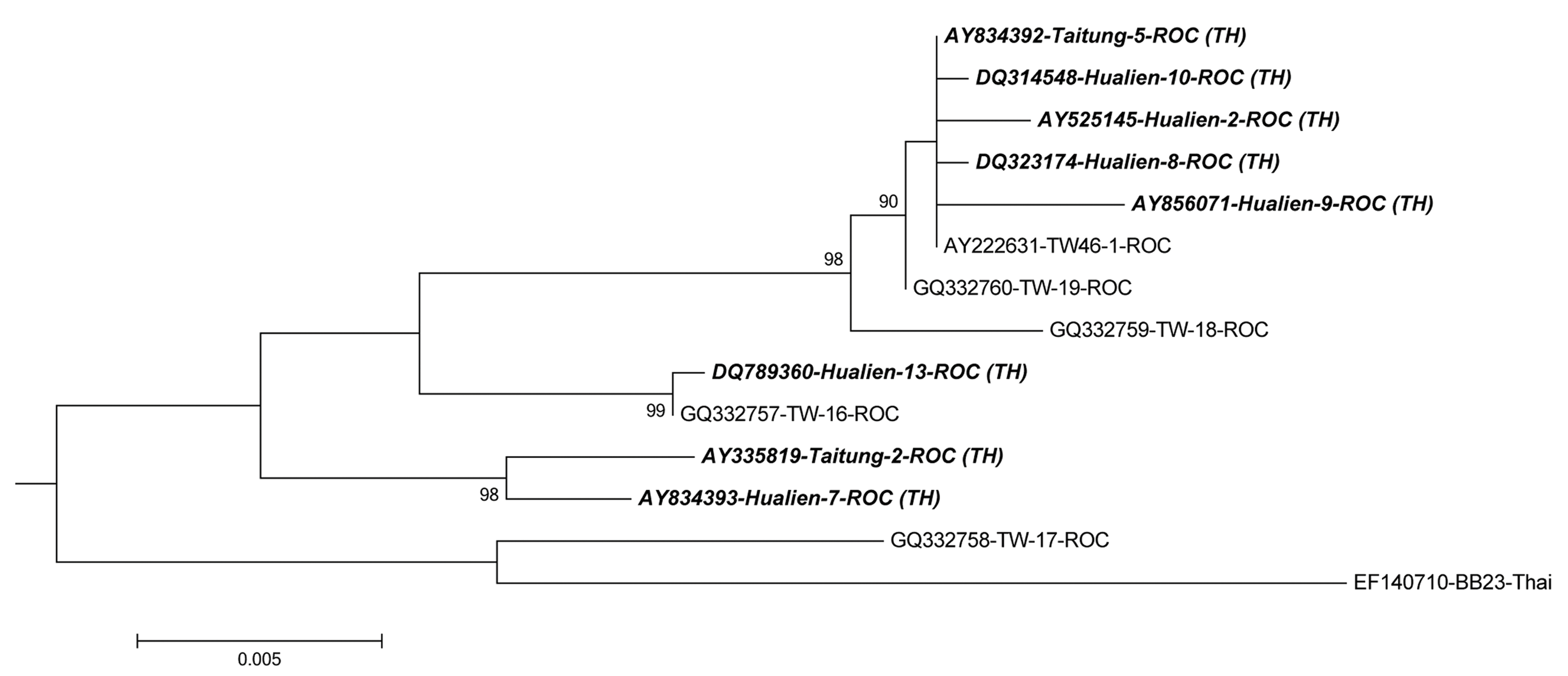

Supplement: Figure S1 — Phylogenetic tree of Taiwan Gilliam-variant cluster based on 56-kDa TSA gene sequences was constructed using a phylogenetic tree of 125 O. tsutsugamushi isolates. TW-16, TW-17, TW-18, and TW-19 are from western Taiwan, TW46-1 is from Taitung Country, and BB23 is from Thailand. The percentage of similarity of the 56-kDa TSA gene sequences among the eight Taiwan-H strains exceeded 98%. (TIF) [file pone.0046997.s001.tif]

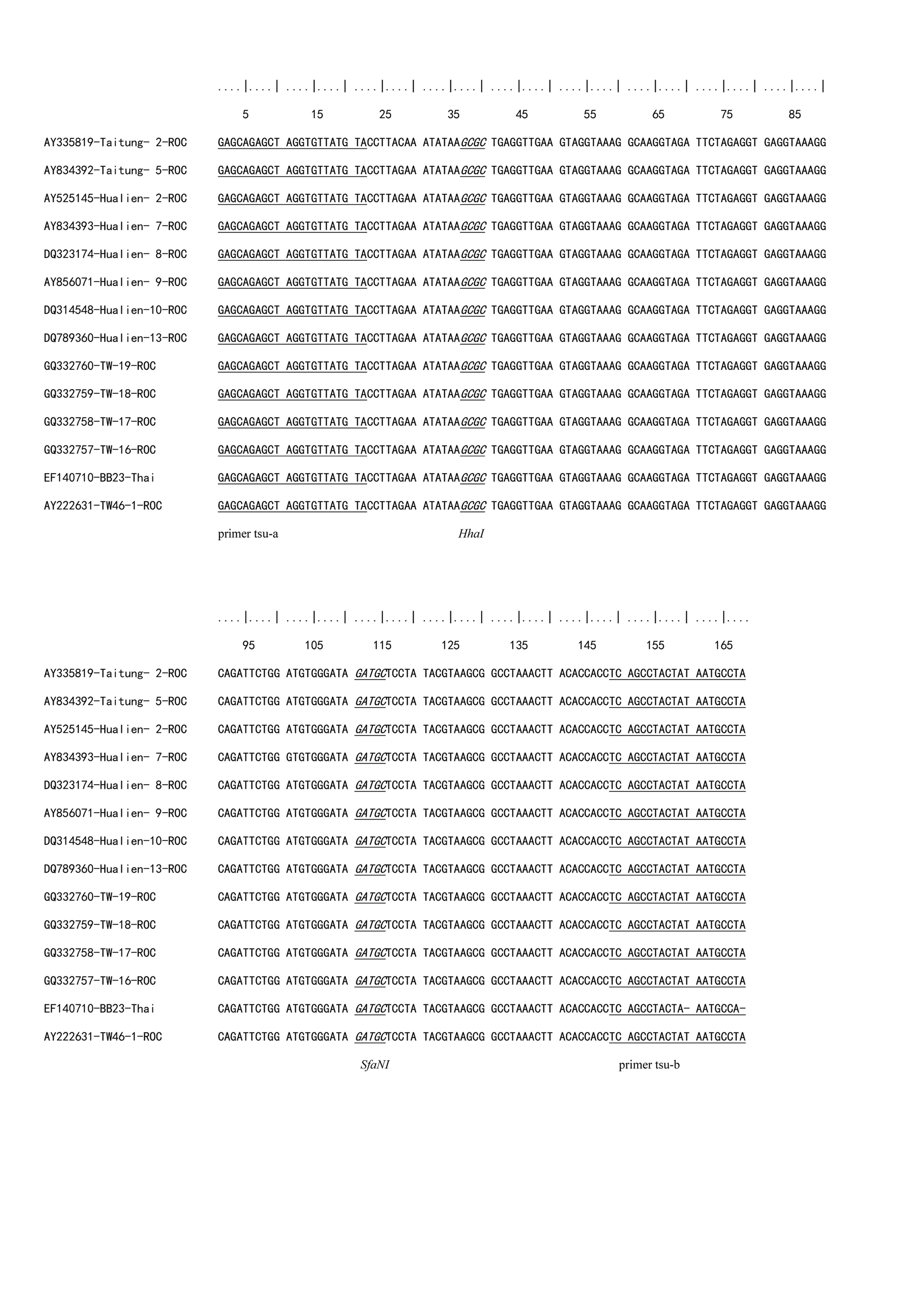

Supplement: Figure S2 — Predicted restriction enzyme sites of the PCR product with primer tsu-a and tsu-b from the TG-v cluster of O. tsutsugamushi isolates. The tsu-a and tsu-b: primer pairs, HhaI and SfaNI : restriction enzymes used in this study. (TIF) [file pone.0046997.s002.tif]

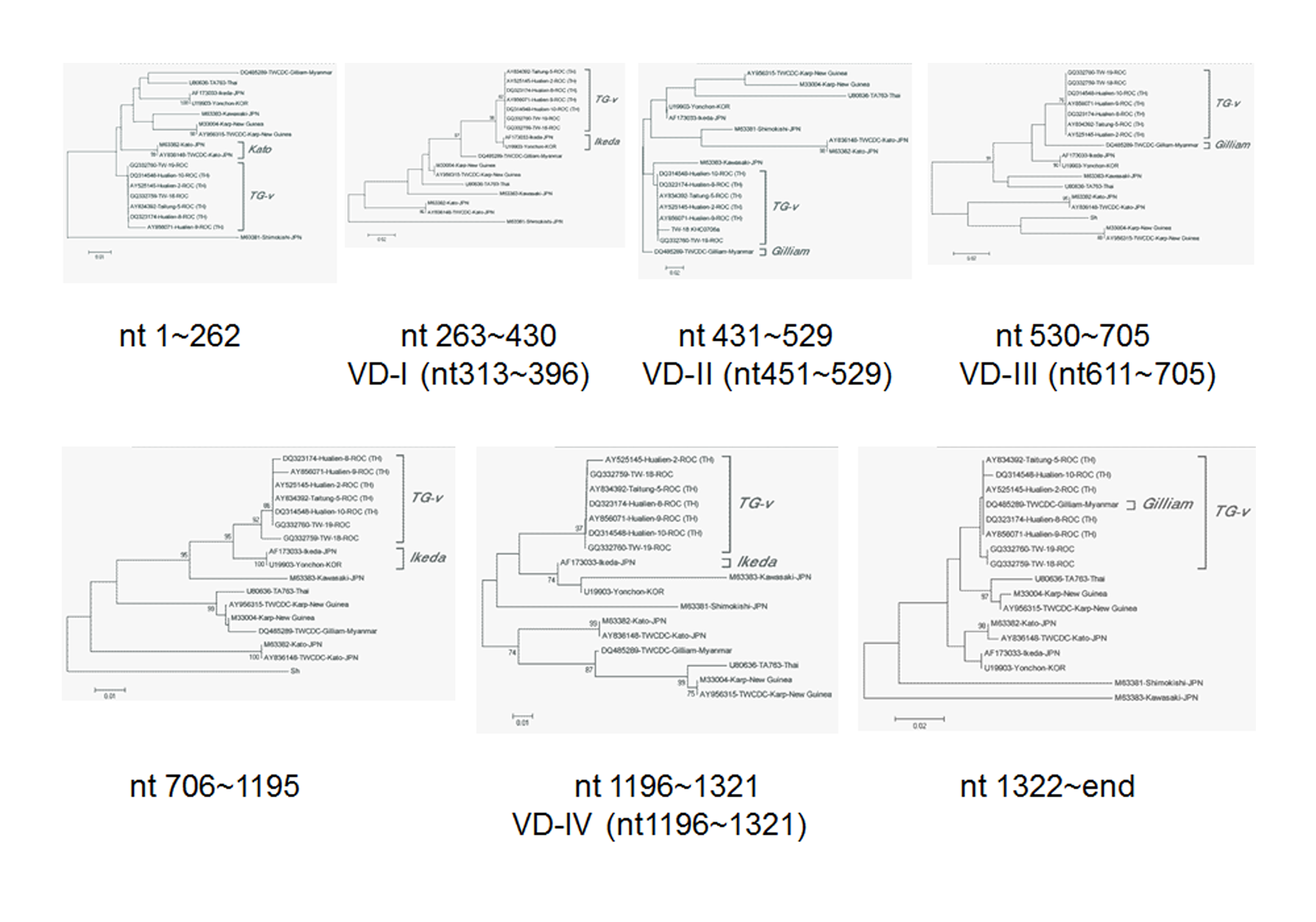

Supplement: Figure S3 — Phylogenetic neighbour-joining trees of different fragments of 56-kDa TSA genes for a selected O. tsutsugamushi isolate (Taitung-5) and other reference strains. (TIF) [file pone.0046997.s003.tif]

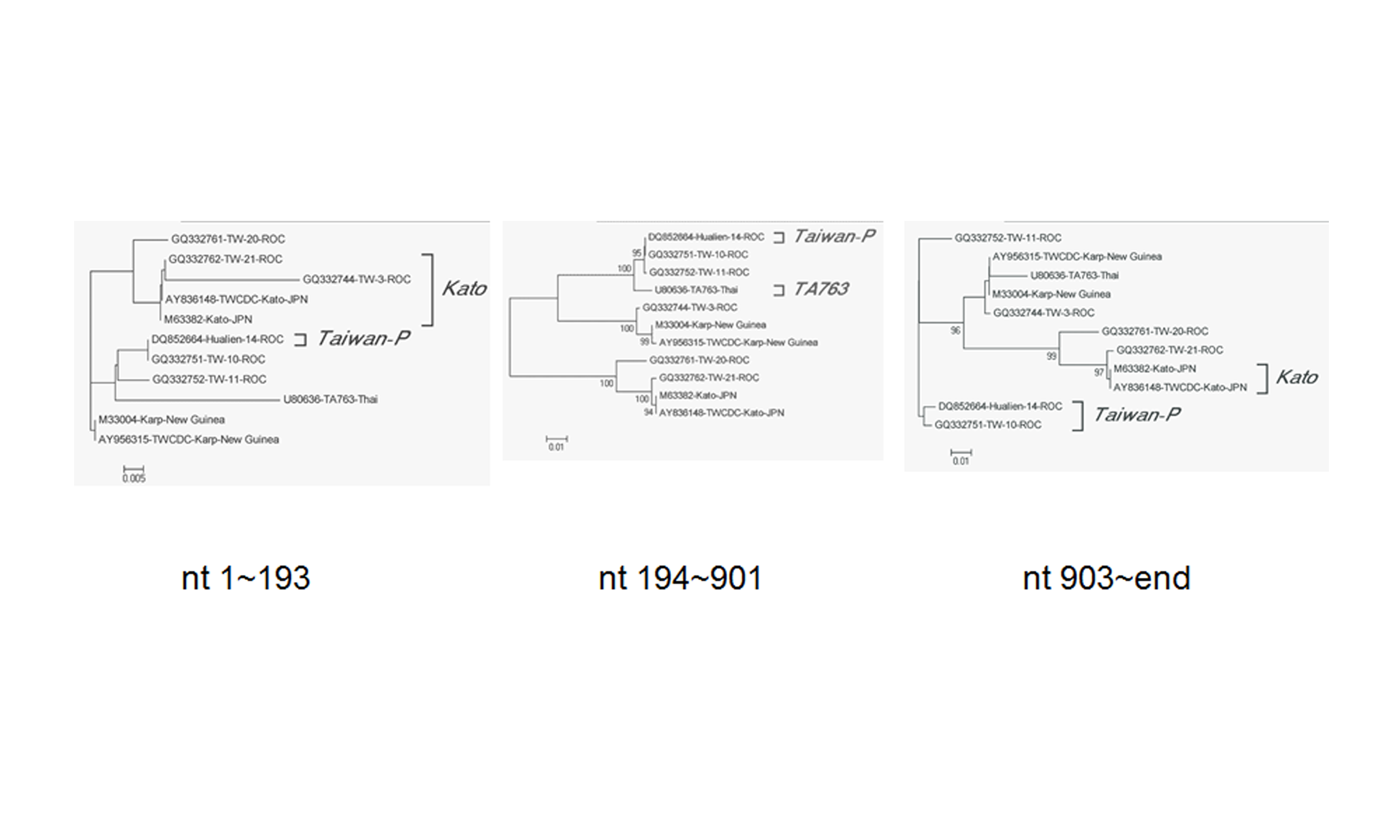

Supplement: Figure S4 — Phylogenetic neighbour-joining trees of different fragments of 56-kDa TSA genes for a selected O. tsutsugamushi isolate (Hualien-14) and other reference strains. (TIF) [file pone.0046997.s004.tif]
